# Right ventricular injury during VV-ECMO for severe ARDS: Does time matter?

**DOI:** 10.1177/17511437251374821

**Published:** 2025-09-24

**Authors:** Robert McDonald, Jo-Anne Fowles, Robert Gatherer, Francisca Caetano

**Affiliations:** 1 School of Clinical Medicine, University of Cambridge, UK; 2 Department of Anaesthesia and Intensive Care, Royal Papworth Hospital NHS Foundation Trust, Cambridge, UK

**Keywords:** right ventricular dysfunction, respiratory failure, acute respiratory distress syndrome, ECMO, venovenous extracorporeal membrane oxygenation

## Abstract

Right ventricular injury (RVI) is a frequent complication during veno-venous extracorporeal membrane oxygenation (VV-ECMO) for severe respiratory failure. In this single-centre retrospective cohort of 40 patients, RVI was observed in 63%, being associated with increased ICU mortality. RVI at admission was more common in younger patients and those with shorter intubation periods pre-cannulation. RVI developing during VV-ECMO was associated with longer ECMO support, ICU stay, and a trend towards higher mortality. The timing of RVI likely reflects different pathophysiology, having different clinical implications. Improved monitoring of right ventricular function during VV-ECMO may enable earlier detection and intervention, potentially improving outcomes.

## Introduction

Right ventricular injury (RVI) is a well-recognised complication in patients with severe acute respiratory distress syndrome (ARDS) of any aetiology and is associated with increased mortality.^1^ The development of RVI is multifactorial, including but not limited to hypoxic pulmonary vasoconstriction, hypercapnic acidaemia, inflammatory mediators, microthrombi and positive pressure ventilation, all of which increase RV afterload.^2^

Venous-venous extracorporeal membrane oxygenation (VV-ECMO) can help alleviate RV afterload through improving gas exchange and allowing for lung-protective ventilation strategies.^3^ Despite the theoretical RV-protective effect of VV-ECMO, RVI may persist or worsen. A recent position statement on behalf of the RVI-ECMO Delphi Expert Group suggested that RVI during VV-ECMO may be subtle initially; however, isolated changes such as RV dilatation may progress and culminate in end-organ hypoperfusion and multiple organ failure.^4^

While RVI is increasingly recognised in this cohort, the timing of its onset – and whether this impacts outcomes and potential clinical management – remains poorly researched.^5^ This study aimed to describe early- and late-onset RVI in patients receiving VV-ECMO for severe ARDS, and to compare clinical features and outcomes between these groups.

## Methods

A single-centre retrospective cohort study of patients admitted to a tertiary centre with severe ARDS requiring VV-ECMO between April 2022 and April 2024. All patients that had at least one echocardiogram (transthoracic (TTE) or transoesophageal (TOE)) performed whilst on VV-ECMO were included.

RVI was defined as the presence of any of the following reported on an echocardiogram: impaired RV function on visual assessment; RV dilatation or right to left ventricular end-diastolic area greater than 0.6 with septal dyskinesia. All scans were reviewed by a BSE Level 2 accredited echocardiographer for consistent classification of RV status.

RVI was classified as ‘early’ if present on the first echocardiogram performed within the first 5 days of ECMO support. Patients that had an initial normal echocardiogram and later developed RVI, were classified as having ‘late RVI’. All echocardiograms included were performed during the ECMO course.

The primary outcome was the timing of RVI (early vs late). Secondary outcomes included ICU mortality, ECMO duration, ICU length of stay and comparisons of clinical characteristics between early and late RVI groups. Statistical analysis was done using Mann-Whitney U and Fisher’s Exact tests using R Studio version 2024.12.1.

## Results

From April 2022-April 2024, 70 patients were admitted to our hospital after being retrieved on mobile VV-ECMO. A total of 40 patients (median age 39.5 years (30, 49); 45% male) met the inclusion criteria for this study (11 were excluded for not fulfilling criteria for severe ARDS, and 19 were excluded due to absence of echocardiographic assessment).

RVI was observed in 25/40 patients (63%) and was associated with higher ICU mortality (12/25 (48%) vs 2/15 (13%), *p* = 0.04).

Of the patients with RVI, 18/25 (72%) had early RVI and the remaining had late RVI. The latter was diagnosed at a median of 46 days after VV-ECMO cannulation (19, 65).

Patients with early RVI were younger (*p* < 0.01), were intubated for fewer days prior to ECMO cannulation (*p* = 0.01), and had a trend to present with higher troponins at admission (*p* = 0.082, Table 1). No significant differences were observed on Murray Score, but a trend to lower RESP score was observed in the late RVI group (*p* = 0.05).

**Table 1.** Characteristics of patients with early versus late right ventricular injury (RVI).

Characteristics	Early RVI (*n* = 18)	Late RVI (*n* = 7)	*p*-value
Age (years)	32 (27, 40)	54 (42, 55)	<0.01
Male sex	5 (28%)	4 (57%)	0.205
Days intubated pre-cannulation	1 (0, 2)	7 (2, 12)	0.012
High sensitivity troponin at admission (ng/L)	358 (166, 976)	56 (20, 301)	0.082
Murray score	3.0 (3.0, 3.8)	3.3 (2.8, 3.3)	0.610
RESP score	4 (3, 5)	2 (−1, 4)	0.052
ICU mortality	7 (39%)	5 (71%)	0.202
ECMO duration (days)	8 (4, 21)	58 (22, 116)	<0.01
ICU length of stay (days)	11 (5, 44)	112 (28, 147)	<0.01

Continuous and ordinal variables are presented as median (lower quartile, upper quartile); categorical variables as count (percentage).

The differences in ICU mortality were notable but not statistically significant - late RVI was linked to longer ECMO support (*p* < 0.01) and longer ICU length of stay (*p* < 0.01) – Table 1.

## Discussion

In this single-centre cohort study, RVI was observed in 63% of patients on VV-ECMO for severe ARDS. In keeping with previous research,^1^ RVI was associated with significantly higher ICU mortality. Two patterns of RVI were observed: early-onset RVI present on the first echocardiogram within the first 5 days of ECMO course, and late RVI, developing later in the VV-ECMO run – up to 65 days into the ECMO course.

Early RVI likely reflects acute RV strain due to pulmonary vasoconstriction, contributed to by hypoxia, hypercapnia, and high ventilator pressures, before extracorporeal support was established.^2^ In contrast, late-onset likely reflects incomplete resolution of ARDS, secondary infective insults and the development of lung fibrosis,^6^ contributing to sustained high RV afterload despite ECMO support.

These findings raise important clinical considerations. While early RVI may be unavoidable in the context of severe ARDS, late-onset RVI could represent a modifiable complication. Previous research^7,8^ has noted the dynamic nature of RVI in this cohort but there is no research which explores the distinct characteristics of early versus late RVI. Currently there is no standardised approach to monitoring right ventricular function on patients supported with VV-ECMO. Implementing routine, structured assessment of RV function throughout the ECMO course may enable earlier detection and hence intervention which might attenuate RV deterioration and improve outcomes.

Limitations of this study include its retrospective design, single centre setting and the small sample size. Echocardiographic assessments were not protocolised, which, combined with the absence of a universally accepted definition of RVI,^5^ may have led to under-recognition or misclassification. The use of inotropes and/or pulmonary vasodilators at the time of echocardiography were not collected which may have also affected classification.

Our findings highlight the need for prospective studies to define optimal timing and methods of RV monitoring, and to determine effective interventions to mitigate RVI during VV-ECMO.
